# Exploring fatty alcohol-producing capability of *Yarrowia lipolytica*

**DOI:** 10.1186/s13068-016-0512-3

**Published:** 2016-05-20

**Authors:** Guokun Wang, Xiaochao Xiong, Rishikesh Ghogare, Pengdong Wang, Yonghong Meng, Shulin Chen

**Affiliations:** Department of Biological Systems Engineering, Washington State University, Pullman, WA 99164-6120 USA; Tianjin Key Laboratory of Industrial Biosystem and Bioprocess Engineering, Tianjin Institute of Industrial Biotechnology, Chinese Academy of Sciences, Tianjin, 300308 People’s Republic of China; College of Food Engineering and Nutritional Science, Shaanxi Normal University, 199 South Chang’an Road, Xi’an, 710062 People’s Republic of China

**Keywords:** Fatty alcohol, Fatty acyl-CoA, Metabolic engineering, Yeast, *Yarrowia lipolytica*

## Abstract

**Background:**

Fatty alcohols are important oleochemicals widely used in detergents, surfactants and personal care products. Bio-synthesized fatty alcohol provides a promising alternative to traditional fatty alcohol industry. Harnessing oleaginous microorganisms for fatty alcohol production may offer a new strategy to achieve a commercially viable yield that currently still seems to be a remote target.

**Results:**

In this study, we introduced functional fatty acyl-CoA reductase (FAR), TaFAR1 to direct the conversion from fatty acyl-CoA to fatty alcohol in *Yarrowia lipolytica* (*Y. lipolytica*), an oleaginous non-conventional yeast showing great lipid-producing capability. Tri-module optimizations including eliminating fatty alcohol degradation pathway, enhancing TaFAR1 expression, and increasing fatty acyl-CoA supply were furtherly conducted, resulting in 63-fold increase in intracellular fatty alcohol-producing capability compared to the starting strain. Thus, this work demonstrated successful construction of first generation of *Y. lipolytica* fatty alcohol-producing cell factory. Through the study of effect of environmental nutrition on fatty alcohol production, up to 636.89 mg/L intracellular hexadecanol (high fatty alcohol-retaining capability) and 53.32 mg/L extracellular hexadecanol were produced by this cell factory through batch fermentation, which was comparable to the highest production of *Saccharomyces cerevisiae* under the similar condition.

**Conclusion:**

This work preliminarily explored fatty alcohol-producing capability through mobilization of FAR and fatty acid metabolism, maximizing the intracellular fatty alcohol-producing capability, suggesting that *Y. lipolytica* cell factory potentially offers a promising platform for fatty alcohol production.

**Electronic supplementary material:**

The online version of this article (doi:10.1186/s13068-016-0512-3) contains supplementary material, which is available to authorized users.

## Background

Fatty alcohols represented a range of aliphatic alcohols with chain lengths ranging from C8 to C32 [1]. Due to their aliphatic character, fatty alcohols find many applications as ingredient of detergents, surfactants, and personal care products [2]. At present, fatty alcohols are mainly produced from petrochemical sources (synthetic fatty alcohols), or derived from renewable resources such as plant or animal-original fats, oils, and waxes (natural fatty alcohols) [3]. Problems derived from these conventional feedstock such as decreasing petroleum supply and competition with food, limited development of fatty alcohol industry [4]. Since the availability of abundant and cost-effective renewable resources for microbe growth, bio-synthesized fatty alcohol provides a promising alternative for traditional fatty alcohol industry.

*Escherichia coli* (*E. coli*) as a prokaryotic model organism, exhibited good capability of producing fatty alcohol. The *E. coli*-mediated fatty alcohol production was realized mainly by redirecting and optimizing metabolic pathway [5, 6]. To confer fatty alcohol-producing capability on *E. coli*, genes coding fatty acyl-CoA reductase (FAR), carboxylic acid reductase (CAR), or fatty acyl-ACP reductase were introduced, driving conversion to fatty alcohol from corresponding metabolite: fatty acyl-CoA, fatty acid, or fatty acyl-ACP [4, 7–10]. *E. coli* strain carrying FAR-encoding gene from *Marinobacter aquaeolei* VT8 and the modified genes for acyl-CoA synthase and thioesterase produced 1.725 g/L fatty alcohols under the fermentation condition [7]. Manipulation of CAR from *Mycobacterium marinum*, aldehyde reductase and chain-length-specific thioesterase made *E. coli* capable to produce more than 350 mg/L fatty alcohol on minimal media supplemented with glucose [10]. Following fatty alcohol-tolerant strain selection, the most productive *E.coli* mutant carrying *Synechococcus elongatus* fatty acyl-ACP reductase produced 0.75 g/L fatty alcohols under fed-batch fermentation with glycerol as the only carbon source [4].

Since the advantage in resistance to phage contamination and the direct availability of fatty acyl-CoA in metabolism [11], eukaryotic model microorganism *Saccharomyces cerevisiae* (*S. cerevisiae*) also gained much attention in bio-synthesized fatty alcohol production. *S. cerevisiae* strain simultaneously overexpressing genes encoding acetyl-CoA carboxylase, fatty acyl-CoA synthase, and *Mus musculus* FAR produced approximately 100 mg/L fatty alcohol after 168 h culturing [11]. Deletion of RPD3, negative regulator in phospholipid metabolism, coupling with overexpression of *Tyto alba* FAR (TaFAR1), acetyl-CoA carboxylase, as well as ATP-dependent citrate lyase allowed *S. cerevisiae* strain to produce 655 mg/L and 1.1 g/L hexadecanol through batch fermentation and fed-batch fermentation, respectively [12]. These studies demonstrated the potential of eukaryote cell factory for fatty alcohol production. Although *E. coli* and *S. cerevisiae* always serve as the conventional cell factories for their easy genetic operation, the model microorganism-based fatty alcohol production is way below the commercially available level. In addition, some drawbacks, mainly associated to the vulnerability to phage infection, the dysfunctional heterologous enzyme production, and insufficient precursor supply, still limited their application in scale production of specific products [13, 14].

Harnessing oleaginous microorganisms for oleochemical production may serve as a new strategy to meet commercially viable yield because of their native potential for lipid production of these organisms. *Yarrowia lipolytica* (*Y. lipolytica*) is an oleaginous non-conventional yeast whose lipid-producing capability has been deeply explored [15–18]. ~55 g/L lipid titer by engineered *Y. lipolytica* strain [18] implicated the abundant metabolic flux to fatty acyl-CoA derivates, as well as the great potential for oleochemical production. As a significant node in cellular oleochemical metabolism, fatty acyl-CoA acts as the precursor for triacylglycerols and sterol synthesis driven by acyl-CoA:diacylglycerol acyltransferase (DGA1-2), phospholipid:diacylglycerol acyltransferase (LRO1), and ACAT-related sterol acyl-CoA acyltransferase (SAT) isozyme (ARE1), respectively [19]. Fatty acyl-CoA was formed through fatty acid activation with the help of fatty acyl-CoA synthetases FAA1 [20, 21], or from acetyl-CoA by the activity of acetyl-CoA carboxylase (ACC) and fatty acid synthase (FAS) [22, 23]. On the other hand, acetyl-CoA was generated from pyruvate-derived acetate or citrate by the activity of acetyl-CoA synthetase (ACS) or ATP-citrate lyase (ACL), respectively [23, 24]. Modestly understood lipid metabolism in *Y. lipolytica* provided a sound platform for oleochemical production, making it the reality of multi-round lipogenesis improvement toward industrial application, however, the capability of producing fatty alcohol of this oleaginous cell factory has not been explored.

**Fig. 1:**
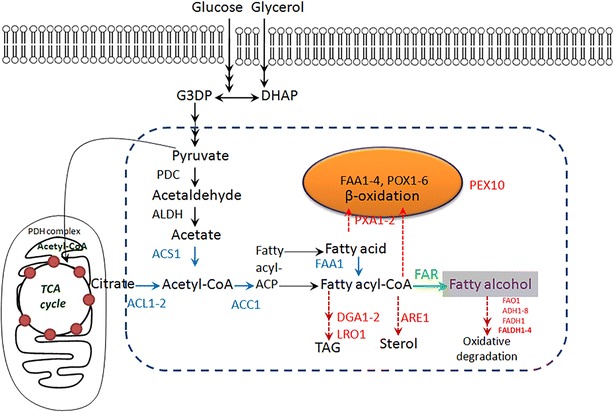
Schematic illustrating the mobilization of *Y. lipolytica* metabolism for fatty alcohol production. Fatty alcohol accumulation was attempted by introducing fatty acyl-CoA reductase (FAR) and eliminating degradation pathways involving fatty alcohol oxidase (FAO), alcohol dehydrogenase (ADH), and fatty alcohol dehydrogenase (FADH). Further improvement of fatty alcohol production was tried by increasing fatty acyl-CoA supply: knock-out of genes responsible for fatty acyl-CoA degradation (transporter PXA and peroxisome biogenesis-involved PEX10) and conversion from fatty acyl-CoA to TAG (acyl-CoA:diacylglycerol acyltransferase DGA and phospholipid:diacylglycerol acyltransferase LRO) or sterol (ACAT-related sterol acyl-CoA acyltransferase (SAT) isozyme ARE); overexpression of genes responsible for directing metabolic flux to fatty acyl-CoA (ATP-citrate lyase ACL, acetyl-CoA synthetase ACS, acetyl-CoA carboxylase ACC, and fatty acyl-CoA synthetase FAA)

In this study, metabolism of *Y. lipolytica* was mobilized to harness this oleaginous microorganism for fatty alcohol production (Fig. 1). Functional FAR, TaFAR1 was introduced to direct the conversion from fatty acyl-CoA to fatty alcohol. *Tafar1* expression strength, degradation pathway of fatty alcohol, and fatty acyl-CoA supply were manipulated to maximize the intracellular fatty alcohol-producing capability, and the first generation of *Y. lipolytica* fatty alcohol-producing cell factory was accordingly constructed. Through effective manipulation of environment especially nutrients for fatty alcohol production, fatty alcohol titer was achieved comparable to the highest production of *S. cerevisiae* through batch fermentation.

## Results

### Fatty alcohol distribution of *Y. lipolytica* with functional fatty acyl-CoA reductase expression

To construct the *Y. lipolytica* cell factory for fatty alcohol production, the relationship between fatty acyl-CoA derivatives and fatty alcohol needs to be established. Fatty acyl-CoA and FAR were selected as the major substrate and catalytic factor for conversion to fatty alcohol in this study. Based on this design, the functional viability of reported FAR-coding genes was tested in *Y. lipolytica*. Strains with episomal expression of six FAR candidates were constructed accordingly.

**Fig. 2:**
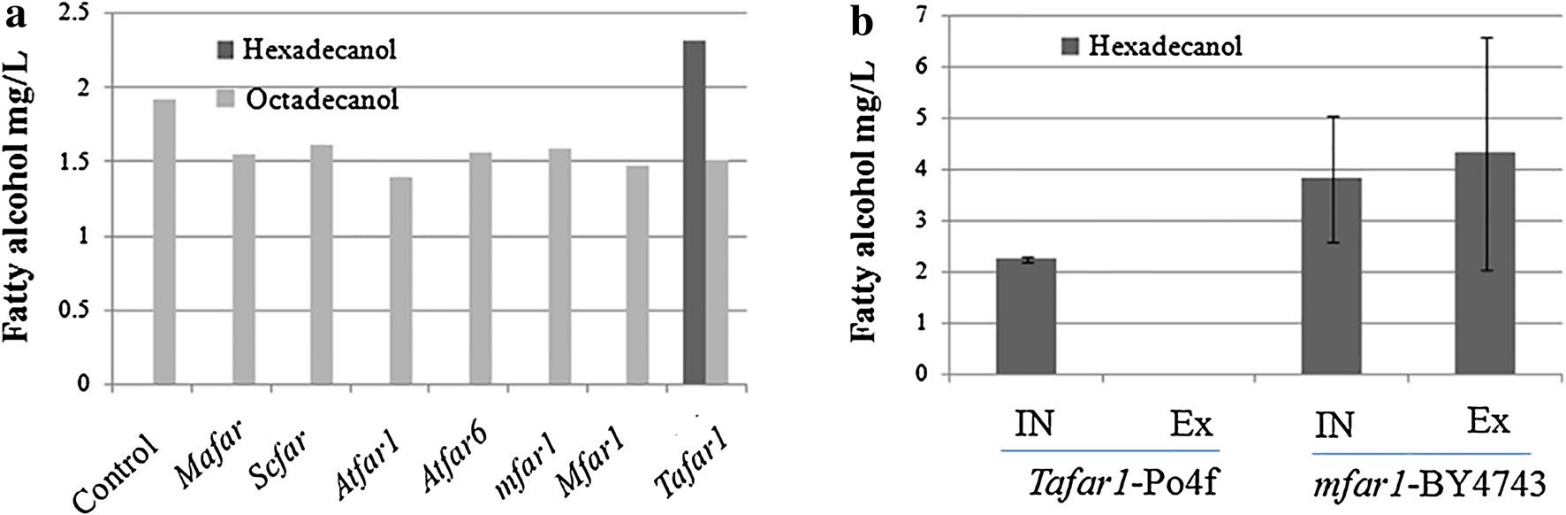
Screening of effective fatty acyl-CoA reductase (FAR)-encoding genes for fatty alcohol production in *Y. lipolytica*. All strains were precultured in SD-LEU medium for 24 h and subcultured to SD-LEU (galactose as carbon source for BY4743) in the initial OD_600_ of 0.05. Detection was performed after 24 h subculturing. **a** Intracellular fatty alcohol amount of Po4f strains expressing FAR gene candidates under the control of TEF promoter. *Mafar*, *Scfar*, *Atfar1*, *Atfar6*, and *mfar1* were obtained through PCR-based cloning, and *Mfar1* (codon optimized version of *mfar1*) and *Tafar1* were codon optimized and synthesized. **b** Comparison of hexadecanol distribution between *Y. lipolytica* and *S. cerevisiae *(IN: intracellular, EX: extracellular). *Tafar1* was driven by TEF promoter while *mfar1* gene was driven by Gal1 promoter. Results are the mean of duplicate experiments and *error bars* indicate standard deviations

All of the strains including wild-type strain (po4f) produced less than 2 mg/L intracellular octadecanol after 24 h culturing (indicated existing of special metabolic pathway of *Y. lipolytica*), whereas only po4f strain expressing FAR from *Barn owl* (*Tafar1*) accumulated hexadecanol (Fig. 2a). This suggested that *Tafar1* encoded functional FAR for in vivo conversion of fatty acyl-CoA to fatty alcohol in *Y. lipolytica*. Large amount of hexadecanol was located extracellular of *S. cerevisiae* strain (Fig. 2b), whereas no hexadecanol was detected in extracellular environment of *Y. lipolytica* strain, demonstrating high capability of retaining fatty alcohol of *Y. lipolytica* cell.

### Eliminating negative effect of degradation pathway on fatty alcohol production

There exist at least thirteen factors contributing to fatty alcohol degradation, including one fatty alcohol oxidase (FAO), eight alcohol dehydrogenase (ADH) [25], and four fatty aldehyde dehydrogenase (FALDH) [26]. To confirm the significance of their negative effect on fatty alcohol production, fatty alcohol-producing capability other than titer of strains lacking corresponding factors was assessed. Since fatty alcohol produced by *Y. lipolytica* was kept inside of the cell and cell growth was retarded by accumulated fatty alcohol (maybe aldehyde, Additional file 1: Figure S1 and Additional file 2: Table S1), fatty alcohol-producing capability was represented with intracellular fatty alcohol amount per unit of cells (OD_600_).

**Fig. 3:**
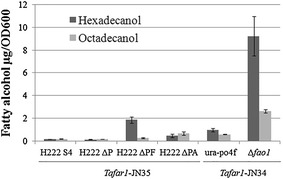
Effect of deleting potential degradation pathways on fatty alcohol-producing capability. In confirming responsible target genes for improving fatty alcohol production, fatty alcohol-producing capability other than fatty alcohol titer was focused and was represented with intracellular fatty alcohol amount per unit of cells (OD_600_), to eliminate growth retarding effect from gene alternations and intracellularly accumulated fatty alcohol/aldehyde, which can be recovered through further adaptive evolution. Knock-out mutants derived from H222S4 or ura-po4f strains expressing *Tafar1* were used for effect evaluation of potential degradation pathways on fatty alcohol-producing capability. After transformation of *Tafar1* expression cassette into corresponding strains, transformants were used for inoculation into SD-URA (H222S4-derived strains) or SD-LEU (ura-po4f-derived strains), and fatty alcohol-producing rate was detected after 24 h culturing. Results are the mean of duplicate experiments and *error bars* indicate standard deviations. H222-S4: *ura3,* H222ΔP: *ura3 pox1*-*6*, H222ΔPΔF: *ura3 pox1*-*6 fao1*, H222ΔPΔA: *ura3 pox1*-*6* *fadh1* *adh1*-*7*

The assessment was firstly conducted on H222-derived strains. As shown in Fig. 3, loss of degradation factors of both categories (fatty alcohol oxidase versus alcohol dehydrogenase) increased fatty alcohol-producing capability. However, the increased margin was significantly higher in strain lacking fatty alcohol oxidase (H222 ΔPF) compared to that without alcohol dehydrogenases (H222 ΔPA). This suggested that fatty alcohol oxidase (FAO1, YALI0B14014g) was the major responsible factor for intracellular fatty alcohol degradation in *Y. lipolytica*. The negative effect of FAO1 was eliminated subsequently in our initial target strain po4f, resulting in ~tenfold increase in the fatty alcohol-producing capability (Fig. 3).

### Effect of *Tafar1* expression strength on fatty alcohol-producing capability

**Fig. 4:**
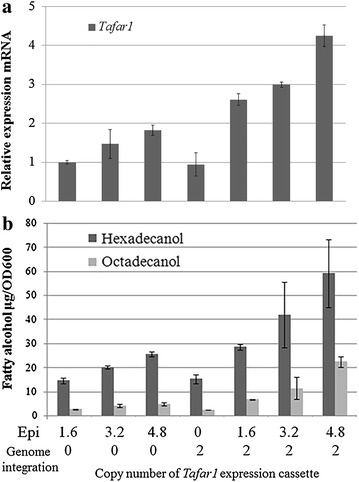
Effect of *Tafar1* expression strength on fatty alcohol-producing capability. Δ*fao1* strains expressing *Tafar1* of different copy numbers were used for effect evaluation of *Tafar1* expression strength on fatty alcohol-producing capability. Low-copy CEN plasmid (~ 1.6 copies/cell) was utilized to control the *Tafar1* copy number (episomal expression, Epi) by manipulating *Tafar1* expression cassette numbers. After transformation of plasmid with different numbers of *Tafar1* expression cassette into Δ*fao1* or *Tafar1*-2copy-Δ*fao1* strains, transformants were obtained (*Tafar1* gene copy number = 1.6 × N + M, where N is *Tafar1* expression cassette number within the plasmid and M is 2 or 0 for strains with or without genome integration of *Tafar1* respectively) and subsequently used for inoculation into SD-LEU. *Tafar1* expression levels (**a**) and fatty alcohol-producing rate (**b**) was detected after 24 h culturing. Relative expression of mRNA was normalized relative to the actin gene and the values reflect fold change expression compared to Po4f uracil + leucine + Tafar1 Epi strain. Real-time PCR results are means of two biological replicates SE. Each PCR was run three times. Results of fatty alcohol producing rate are the mean of duplicate experiments and *error bars* indicate standard deviations

FAR is the key catalytic factor to drive the metabolic flux from fatty acyl-CoA to fatty alcohol, hence its intracellular amount is supposed to be decisive to the quota of fatty acyl-CoA to fatty alcohol, as well as the rate achieving the reaction balance. To determine the contribution of FAR amount on fatty alcohol production, *Tafar1* expression strength was manipulated by controlling the *Tafar1*’s copy number, and *Y. lipolytica* Δ*fao1* strains with different *Tafar1* expression levels were generated (Fig. 4 and Additional file 1: Figure S2 and S3).

Manipulation of *Tafar1* gene copy number was achieved by controlling the number of *Tafar1* expression cassette both located dependently (genome integration of two-copy cassette, URA3 marker) and independently (episomal plasmids with different cassette numbers, LEU2 marker) of the chromosome (Fig. 4). The expression plasmids used in this study were low-copy CEN plasmids (1–2 copies/cell [27], ~1.6 plasmid copies/cell [28]), allowing us to gradually increase *Tafar1*’s expression level by the combinatorial manipulation of episomal and stable genome-derived expressions (Fig. 4a).

Fatty alcohol-producing capability elevated with the increase in copy number of *Tafar1* expression cassette (the elevation was medium independent (Additional file 1: Figure S2), achieving up to 73.19 µg hexadecanol per OD_600_ cells (Fig. 4b). This suggested that fatty alcohol production was tightly dependent on the expression strength of FAR and highly expressed *Tafar1* is prerequisite for high production of fatty alcohol.

### Effect of fatty acyl-CoA supply on fatty alcohol-producing capability

**Fig. 5:**
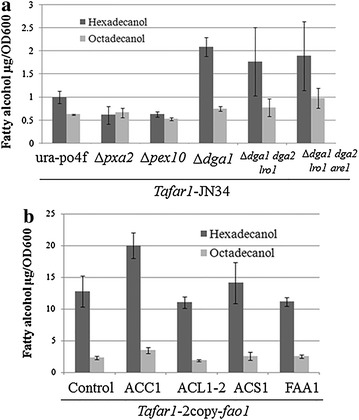
Effect of fatty acyl-CoA supply on fatty alcohol-producing capability. Fatty alcohol-producing rate was detected in knock-out strains expressing *Tafar1* (**a**) and *Tafar1*-2copy-Δ*fao1* strains expressing genes introducing carbon flux to fatty acyl-CoA (**b**). Transformants were used for inoculation into SD-LEU and fatty alcohol-producing rate was detected after 24 h culturing. Results are the mean of duplicate experiments and *error bars* indicate standard deviations

As the direct substrate for the conversion to fatty alcohol by FAR, fatty acyl-CoA was supposed as a key component and its amount was speculated as a limiting factor for fatty alcohol production. To test this hypothesis and confirm the potential target for improvement in fatty alcohol-producing capability, strains were generated lacking competing pathways of fatty acyl-CoA or expressing genes directing metabolic flux to fatty acyl-CoA. Loss of transporter PXA2 (YALI0D04246g) or peroxisome biogenesis-involved PEX10 (YALI0C01023g) slightly decreased the hexadecanol-producing capability, whereas deleting DGA1 (YALI0E32769g) elevated the fatty alcohol-producing capability by twofold (Fig. 5a). Further knocking out DGA2 (YALI0D07986g), LRO1 (YALI0E16797g), and ARE1 (YALI0F06578g) did not significantly increase the fatty alcohol-producing capability under the condition used in this study (Fig. 5a). This indicated that DGA1 was mainly responsible for the competition of fatty acyl-CoA with FAR.

Overexpression of *Y. lipolytica* ACL (YALI0E34793g and YALI0D24431), FAA1 (YALI0D17864g), and *S. cerevisiae* ACS1 failed to increase the fatty alcohol-producing capability (Fig. 5b). Unlikely, elevated expression of ACC1 (YALI0C11407g) resulted in ~1.5-time increase in fatty alcohol-producing capability (Fig. 5b) with severe negative side effect on cell growth (data not shown).

### Dependency of hexadecanol production of combinatorially engineered strain on culturing condition

To thoroughly explore *Y. lipolytica*’s capability of producing intracellular fatty alcohol, combinatorial engineering of above useful targets and culture process optimization were performed. Although deleting *dga2*, *lro1*, and *are1*, as well as overexpressing *acc1* was favorable for the fatty alcohol-producing capability, such manipulations significantly repressed cell growth (data not shown) thus were omitted in our final design of *Y. lipolytica* fatty alcohol-producing cell factory. As a result, *Tafar1*-5copy-Δ*dga1fao1* strain (NO. 20 strain in Additional file 1: Figure S4 B) was generated as the first generation *Y. lipolytica* cell factory for fatty alcohol production. This strain showed ~63-fold increase in the fatty alcohol-producing capability compared to the starting strain (Po4f uracil + leucine + Tafar1 Epi) (54.25 VS 0.8607 µg hexadecanol per OD_600_ cells).

Carbon source supply or C/N ratio was reported to significantly affect the lipogenesis induction of *Y. lipolytica* [16, 29]. Effects of carbon source supply and C/N ratio on fatty alcohol (oleochemical whose position is similar with lipid in the metabolic pathway) production were thus confirmed for the purpose of increasing fatty alcohol production.

**Fig. 6:**
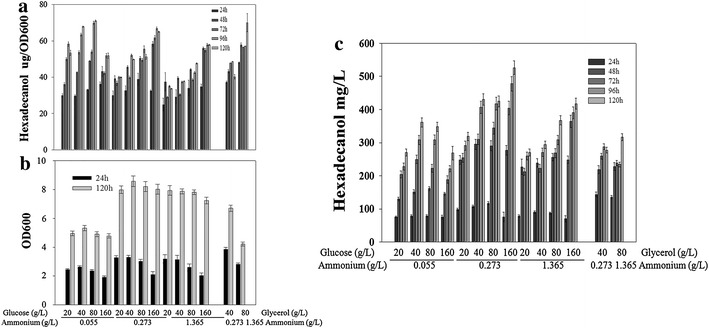
Dependency of *Tafar1*-5copy-Δ*dga1* *fao1* strain’s growth and intracellular hexadecanol production on carbon source and C/N ratio. Hexadecanol-producing capability (**a**), cell growth (**b**), and hexadecanol production (**c**) of the engineered *Tafar1*-5copy-Δ*dga1* *fao1* strain in basal SD medium with altered combination of carbon and nitrogen source were detected over 5 days. Pre-culture of the strain (SD medium) was inoculated into fourteen medium formulations containing glucose/glycerol (between 20 and 160 g/L) and ammonium (between 0.055 and 1.365 g/L) in the initial OD_600_ of 0.05 for cultivations and detections. These studies were conducted twice and results of one batch were presented

Hexadecanol-producing rate (fatty alcohol-producing capability) increased over cultivating time, achieving a stable level after 48 h (groups with 0.273 or 1.365 g/L ammonium) or 96 h (group with 0.055 g/L ammonium) culturing (Fig. 6a). The hexadecanol-producing rate was higher in cells cultured on medium with relatively high C/N ratio, demonstrating the highest intracellular hexadecanol-producing rate of 71.41 µg hexadecanol per OD_600_ cells (Fig. 6a, 80 g/L glucose and 0.055 g/L ammonium). Extremely high C/N ratio might be responsible for the lower hexadecanol-producing rate of cells on medium with 160 g/L glucose and 0.055 g/L ammonium (Fig. 6a). Although high C/N ratio was favorable for hexadecanol-producing capability, insufficient ammonium supply limited cell growth: final cell amount was significantly lower on medium with 0.055 g/L ammonium compared to those with 0.273 or 1.365 g/L ammonium (Fig. 6b). As intracellular fatty alcohol production relied on both fatty alcohol-producing capability and cell number, the highest final hexadecanol production was achieved on medium with 160 g/L glucose and 0.273 g/L ammonium, reaching 546.57 and 636.89 mg/L hexadecanol after 120 h and 144 h batch culturing (Fig. 6c; Table 1). 53.32 mg/L extracellular hexadecanol was also detected after 144 h culturing (Table 1).

**Table 1:** Hexadecanol titer of *Tafar1*-5copy-Δ*dga1* *fao1* strain after 144 h cultured on medium with 160 g/L glucose and 0.273 g/L ammonium

Distribution	Intracellular	Extracellular
Titer (mg/L)	610.50 ± 26.39	50.32 ± 3.00

Glycerol was reported to repress transcription of genes involved in the assimilation of alkanes and fatty acids in *Y. lipolytica* [30]. Since these genes may participate in the fatty alcohol metabolism of our cell factory, the possibility of increasing fatty alcohol production with glycerol as carbon source was also tested. When cultured on medium with glycerol as sole carbon source, cells showed higher hexadecanol-producing rate, biomass accumulation, and resultant hexadecanol production at the early culturing stage (24 h, Fig. 6a–c), nevertheless, they did not show advantage on final hexadecanol production (Fig. 6c).

## Discussion

Fatty alcohol production through engineered cell factory represents a promising approach less dependent on the decreasing petroleum supply and food-associated feedstock. For the purpose of constructing fatty alcohol-producing cell factory, many efforts have been made with *E. coli*, cyanobacteria, and *S. cerevisiae* [4, 6–8, 10–12, 31, 32], and up to 1.725 g/L fatty alcohol production was achieved [7]. Although *E. coli* and *S. cerevisiae* serve as sound model microorganisms for cell factory construction, titers of lipid-derived products by engineered *E. coli* or *S. cerevisiae* are usually incomparable to that of wild-type oleaginous microorganisms [11, 16, 33, 34]. This means oleaginous microorganism has greater potential for bio-synthesized oleochemical production because of their basal lipid accumulation. Specifically, an oleaginous microorganism, *Y. lipolytica* has been developed as a platform for lipid and biofuel production [16], and ~55 g/L lipid titer was achieved by engineered *Y. lipolytica* strain [18]. In the current work, capability of *Y. lipolytica* producing fatty alcohol was explored through mobilization of fatty acid metabolism (Fig. 1) and culturing optimization, toward the commercially viable level of bio-synthesized fatty alcohol.

TaFAR1 [35], the sole functional FAR of those tested in *Y. lipolytica*, was utilized to convert fatty acyl-CoA to fatty alcohol. Compared to CAR and fatty acyl-ACP reductase, products of which are fatty aldehyde [4, 10, 36], FAR was responsible for the direct conversion of fatty acyl-CoA to fatty alcohol through the intermediate of fatty aldehyde. In addition, FARs were the most widely used enzymes for construction of fatty alcohol-cell factory because of their high efficiency, and the highest titer was also achieved by *Rhodosporidium toruloides* fatty alcohol-producing cell factory expressing *Mafar* until now [37]. Hence FAR was selected for connection of fatty alcohol with intracellular fatty acid metabolism of *Y. lipolytica*, and *Y. lipolytica* fatty alcohol-producing cell factory was accordingly constructed.

Fatty alcohol produced by *E. coli* and *R. toruloides* was mainly secreted extracellularly [4, 37], and the secreted fatty alcohol by *S. cerevisiae* was also detectable [11, 38]. Contrast to this, all fatty alcohol produced by *Y. lipolytica* was kept inside of the cells when the production was low (Fig. 2b), indicating the high fatty alcohol retention capability [maximized intracellular hexadecanol: 71.41 to 73.19 µg hexadecanol per OD_600_ cells (Figs. 4, 6)]. This was understandable since *Y. lipolytica* serves as an oleaginous microorganism capable to utilize hydrophobic substrates such as alkane and lipids [23]. This character is essential and favorable for the retainment of nutrition for *Y. lipolytica* growth, and may be derived from special storage mechanism and incompetent outward transport of intracellular hydrophobic substrates. On one hand, this is undesirable for construction of cell factory for oleochemical production as secretion of the product is preferable. On the other hand, this provided us the opportunity to maximize the intracellular fatty alcohol production for the construction of the first generation fatty alcohol-producing *Y. lipolytica* cell factory.

Since fatty alcohol was derived from fatty acyl-CoA by the activity of FAR and was mainly kept inside of the cells, cell can be regarded as a close reactor for multi-module optimization toward maximized intracellular fatty alcohol production. Total intracellular fatty alcohol titer relied on the cell number and fatty alcohol-containing amount per cell. Since fatty alcohol generation resulted from the enzymatic catalysis from fatty acyl-CoA by TaFAR1 [35], the amount of fatty alcohol (product) was dependent on the product accumulation, amount of TaFAR1 striving for fatty acyl-CoA (substrate), and the enzymatic balance, as well as fatty acyl-CoA (substrate) supply. Tri-module optimizations were accordingly conducted: eliminating fatty alcohol degradation pathway, enhancing TaFAR1 expression, and increasing fatty acyl-CoA supply. Following identification and manipulation of available targets, up to 63-fold increase in fatty alcohol-producing capability was achieved after 24 h culturing (Fig. 3 and Additional file 1: Figure S4). Among the ten components involved in oxidative fatty alcohol degradation essential for alkane metabolism [25], FAO1 was the most responsible factor, deletion of which increased fatty alcohol-producing capability by ~10 times (Fig. 3). TaFAR1 expression level was also decisive to the fatty alcohol-producing capability, optimization of which resulted in ~fourfold increase (Fig. 4). Blocking fatty acyl-CoA to triacylglycerols by *dga1* deletion and overexpressing *acc1* resulted in 1.5 to 2-fold increase in fatty alcohol-producing capability (Fig. 5), this was similar to previous studies [11, 38] and indicated that *dga1* deletion and *acc1* overexpression elevated the fatty acyl-CoA amount to provide more substrate for conversion to fatty alcohol. Contrast to combinatorial positive effects of ACC1 and ACL on fatty alcohol production in *S. cerevisiae* [12], increased acetyl-CoA by overexpressing ACL and *S. cerevisiae* ACS1 [39] failed to directly increase fatty acyl-CoA without *acc1* overexpression (Fig. 5). Deletion of PXA2 or POX1 had no obvious impact on fatty alcohol production in *S. cerevisiae* [11], unlike to this, loss of peroxisome-related genes (*pxa2*, *pex10*, *pox1*-*6*) decreased the fatty alcohol-producing capability (Figs. 3, 5), implying the peroxisome’s special role in fatty acyl-CoA regeneration in *Y. lipolytica*.

Fatty alcohol production by *Y. lipolytica* was also dependent on the environmental factors (Fig. 6). High C/N ratio represses isocitrate dehydrogenase activity and ensures sufficient citrate acid supply for acetyl-CoA and subsequent lipid metabolism [29]. Recent study identified that lipid synthesis of *Y. lipolytica* was ultimately controlled by carbon amount and was dependent on leucine-mediated signaling [16]. Nitrogen-permissive and high-carbon conditions are optimally suitable for highly lipogenic strains’ lipid accumulation [16]. In the case of fatty alcohol, fatty alcohol-producing capability of *Y. lipolytica* was independent on leucine-mediated signaling (Additional file 1: Figure S3) and was highly correlated to the C/N ratio (Fig. 6). Another key factor for the intracellular fatty alcohol titer, high cell number, was achieved and maintained by sufficient carbon and nitrogen supplies (Fig. 6). Hence both moderately high C/N ratio and adequate carbon and nitrogen supply contributed to the fatty alcohol production of *Y. lipolytica*. Glycerol as alternative carbon source, was advantageous in faster accumulation of *Y. lipolytica* cells (Fig. 6) thus offered an approach for increasing the productivity by carbon source optimization. Since fatty alcohol titer is biomass-dependent, utilization of enriched media for *Y. lipolytica*-based fatty alcohol production may be more promising than supportive media (used in this study) for the advantage in biomass accumulation.

## Conclusion

First generation fatty alcohol-producing *Y. lipolytica* cell factory was constructed by connecting fatty alcohol with fatty acyl-CoA, mobilization of fatty acid metabolism, and culturing optimization. Up to 636.89 mg/L intracellular hexadecanol and 53.32 mg/L extracellular hexadecanol was produced by this cell factory through batch fermentation. The titer was comparable to the highest fatty alcohol production by *S. cerevisiae* under batch fermentation. Since the titer was obtained from *Y. lipolytica* strain of which only fatty acid metabolism was manipulated, this work suggested that *Y. lipolytica* cell factory exhibited a potential for fatty alcohol production. The highest yield of the first generation *Y. lipolytica* cell factory was 0.018 g/g, far below the theoretical yield (~0.34 g/g, value of *S. cerevisiae* [12]). Further improvements would be releasing fatty alcohol’s (product) inhibition on the enzymatic reaction catalyzed by TaFAR1 (reducing fatty alcohol-retaining capacity), and eliminating redundant energy-consuming pathways.

## Methods

### Strains and culture condition

*Escherichia coli* top 10 was used as the host strain for plasmid construction and propagation. The *Y. lipolytica* strains used in this study were all derived from Po1f (ATCC MYA-2613) or H222 [40]. *S. cerevisiae* strain BY4743 was used for positive control of fatty alcohol producer. All strains used in this study are listed in Additional file 2: Table S2.

Complete Synthetic Defined Media (SD) contains 20 g/L glucose, 6.7 g/L yeast nitrogen base (YNB) w/o amino acids [5 g/L (NH_4_)_2_SO_4_, and 1.7 g/L YNB, Becton, Dickinson and Company], and 0.79 g/L complete supplement mixture (CSM). SD-URA, in which CSM was replaced by drop-out mix synthetic minus uracil (2 g/L), and SD-LEU (minus leucine) were used for transformants’ selection and corresponding strains’ culturing (for fatty alcohol detection). Glucose was substituted with galactose for BY4743-derived strains. 20 g/L agar was added for solid plate preparation. Yeast peptone dextrose (YPD) medium was used as enriched medium to confirm the independence of improvement in fatty alcohol-producing capability on medium type and construction of correlation between optical density (OD_600_) and dry cell weight (DCW) (Additional file 1: Figure S5).

For fermentation medium optimization (C/N ratio), the reported medium formulation was used [16], containing 1.7 g/L YNB w/o amino acids and (NH_4_)_2_SO_4_, 0.79 g/L CSM, glucose (20, 40, 80 or 160 g/L), and (NH_4_)_2_SO_4_ (0.2, 1 or 5 g/L). For test of glycerol as carbon source, glucose was substituted with glycerol in corresponding medium [40 g/L glucose + 1 g/L (NH_4_)_2_SO_4_; 80 g/L glucose + 5 g/L (NH_4_)_2_SO_4_].

### Plasmid construction

*Y. lipolytica* plasmids pJN34 (P_TEF_-Txpr2), pJN35 (P_TEF_-Txpr2), pJN44 (P_TEFin_-Txpr2), pGR13 (P_FBA_-Tlip1), and pGR53 (P_GPM_-Toct1) were used for gene expression in this study. They are centromeric, replicative vector with leucine selection marker except pJN35 (uracil selection marker).

Gene segments of fatty acyl-CoA reductase were obtained by polymerase chain reaction (PCR) plasmids requested from elsewhere (*Marinobacter aquaeolei Mafar*, *Simmondsia chinensis Scfar*, *Arabidopsis thalianaAtfar1*, *Atfar6,* and *Mus musculus mfar1*) as templates with primers listed in Additional file 2: Table S3. The PCR products or synthesized gblock (*Barn owlTafar1* and *Mus musculusMfar1*, both condon optimized) were digested, purified, and subcloned into the pJN34 expression vector.

Gene segments of *Ylacc1*, *Ylacl1*, *Ylacl2*, *Ylfaa1,* and *Scacs1*, encoding acetyl-CoA carboxylase (ACC), ATP-citrate lyase (ACL), fatty acyl-CoA synthetase (FAA), and acetyl-CoA synthetase (ACS), were obtained by PCR using genome DNA as templates. The PCR products were digested, purified, and subcloned into pJN44, pGR53, pGR13, pGR13, and pGR53 respectively. pGR53 and pGR13 are plasmids with same construction as pJN44 varying with promoters and terminators.

For construction of plasmid with *acl1*-*acl2* expression cassette, segment of P_FBA_-*Ylacl2*-Tlip1 was obtained by digestion with XbaI and SpeI, and was inserted into SpeI and Fast Alkaline Phosphatase digested *Ylacl1*–pGR53 plasmid. Construction of plasmid with *Tafar1* expression cassette of various copies was performed similarly.

Plasmids for gene knock-out contained the uracil selection marker surrounded by LoxP sites. For knock-out plasmid construction, the 5′ and 3′ flanking regions of corresponding genes were amplified with the primers listed in Additional file 2: Table S3, digested, purified, and inserted into the upstream and downstream of uracil selection marker, respectively.

### Strain construction

Episomal expression plasmids were used for transformation toward screening of responsible fatty acyl-CoA reductases, as well as effect assessment of degradation pathways, *Tafar1* expression strength and fatty acyl-CoA supply on fatty alcohol production. Combinatorial construction of high-efficiency fatty alcohol-producing strain (*fao1* uracil + *Tafar1*-*2* leucine- and *dga1 fao1* uracil + *Tafar1*-*2* leucine + *Tafar1*-*3*; random insertion) and knock-out strains (homologous recombination) were achieved by transformation with linearized plasmids constructed as presented above. Transformation was performed with Zymogen Frozen EZ yeast transformation kit II (Zymo Research Corporation) according to the manufacturer’s instruction.

Knock-out mutants were constructed through multiple-round homologous recombination (transformation with linearized knock-out cassette) and marker rescue (Cre-Recombinase based uracil marker deletion) as previously described [41].

### RNA isolation and transcript quantification

For *Tafar1* expression level evaluation, 24 h subculture of strains expressing *Tafar1* was collected and subjected to RNA extraction using AllPrep DNA/RNA mini kit (Qiagen) following the manufacturer’s instruction. Specially, cell lysis was performed according to previous study [17]. RNA was reverse transcribed into cDNA with SuperScript Reverse Transcriptase (Invitrogen). Transcript quantification (qRT-PCR) was performed using PowerUp SYBR Green Master Mix (Applied Biosystems) according to the manufacture’s instruction. Actin (YALI0D08272g) was amplified as a loading control and all PCRs were performed in triplicate.

### Fatty alcohol extraction and quantification

For assessment of strains’ fatty alcohol-producing capability with episomal expression plasmid, three transformants’ colonies were used for inoculation of each strain. Among these strains, preculturing and subculturing (initial OD_600_ of 0.05) of strains expressing FAR on SD-LEU were performed for screening of responsible FAR, whereas only preculturing was performed before fatty alcohol detection for other strains with episomal expression plasmids. Preculturing was performed in culture tubes containing 2.5 mL of corresponding selective SD media. 250 mL Erlenmeyer flasks with 50 mL of corresponding SD medium was used for subculturing. The fermentation was carried out at 30 °C on rotary shaker at 180 rpm. Fatty alcohol was extracted and detected at 24 h for both precultures and subcultures.

For determination of engineered strain’s (*Tafar1*-5copy-Δ*dga1 fao1*) fatty alcohol-producing capability, preculturing on SD-LEU and subculturing (initial OD_600_ of 0.05) on medium with various carbon and nitrogen contents were performed. The incubation procedure was same as above and fatty alcohol detection was performed every 24 h after subculturing. Culturing and fatty alcohol quantification of *S. cerevisiae* cells were performed as previously described [12].

Culture sample was taken for fatty alcohol detection. Following measurement of optical density at 600 nm, 1 mL culture was subject to centrifugation at 14,000*g* for 5 min. Supernatant was used for extracellular fatty alcohol extraction with ethyl acetate of same volume after another round centrifugation, whereas cell pellet was resuspended with ethyl acetate and disrupted using glass beads for 5 min. After centrifugation at 14,000*g* for 5 min, supernatant was collected for quantification with GC-FID. Fatty alcohol analysis with GC-FID was performed as previously described [4].

## Additional files

10.1186/s13068-016-0512-3 GC-FID analysis of fatty alcohol samples extracted from engineered strains (A) and growth inhibition of fatty alcohol accumulation (B). **Figure S2.** Fatty alcohol-producing capability of engineered strains on enriched medium (YPD). **Figure S3.** Limited positive effect of leucine supplement on fatty alcohol production by *Tafar1*-2copy-Δ*fao1* strain. **Figure S4.** Two-round screening (A and B) and purification of *Tafar1*-5copy-Δ*dga1* *fao1* strains. **Figure S5.** The correlation between optical density (OD_600_) and dry cell weight (DCW) of *Y. lipolytica* cells.
10.1186/s13068-016-0512-3 Demonstration of growth retardation from fatty alcohol/aldehyde accumulation in 24 h. **Table S2.** Strains used in this study. **Table S3.** Primers used in this study.

## Abbreviations

ACC: acetyl-CoA carboxylase
ACL: ATP-citrate lyase
ACS: acetyl-CoA synthetase
ARE1: ACAT-related sterol acyl-CoA acyltransferase isozyme
CAR: carboxylic acid reductase
CSM: complete supplement mixture
DGA: acyl-CoA:diacylglycerol acyltransferase
FAA: fatty acyl-CoA synthetase
FAR: fatty acyl-CoA reductase
FAS: fatty acid synthase
LRO: phospholipid:diacylglycerol acyltransferase
SD: synthetic defined media
YNB: yeast nitrogen base
