# Supplementary material for: Exploring fatty alcohol-producing capability of Yarrowia lipolytica
Source: Biotechnol Biofuels. 2016 May 20;9:107. doi: 10.1186/s13068-016-0512-3 (PMC4875687; doi:10.1186/s13068-016-0512-3)
Supplement: Supplementary file 1 — 10.1186/s13068-016-0512-3 GC-FID analysis of fatty alcohol samples extracted from engineered strains (A) and growth inhibition of fatty alcohol accumulation (B). Figure S2. Fatty alcohol-producing capability of engineered strains on enriched medium (YPD). Figure S3. Limited positive effect of leucine supplement on fatty alcohol production by Tafar1-2copy-Δfao1 strain. Figure S4. Two-round screening (A and B) and purification of Tafar1-5copy-Δdga1 fao1 strains. Figure S5. The correlation between optical density (OD600) and dry cell weight (DCW) of Y. lipolytica cells. [file 13068_2016_512_MOESM1_ESM.pdf]

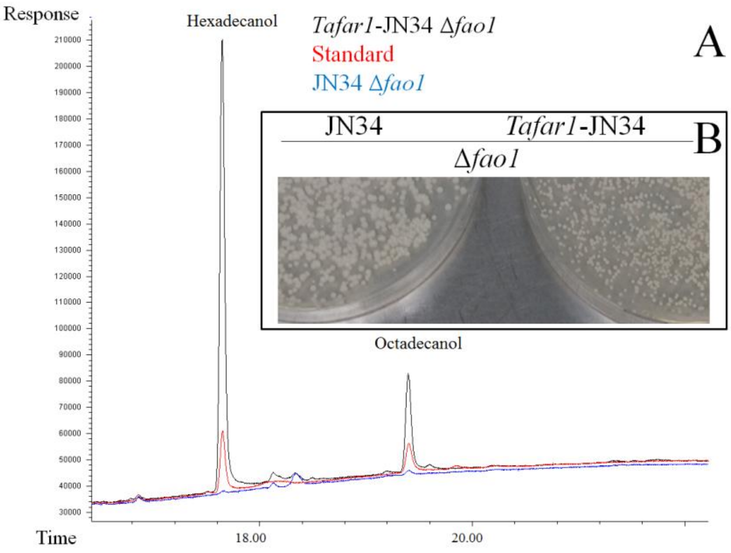

Fig. S1 GC-FID analysis of fatty alcohol samples extracted from engineered strains (A) and growth inhibition of fatty alcohol accumulation (B)

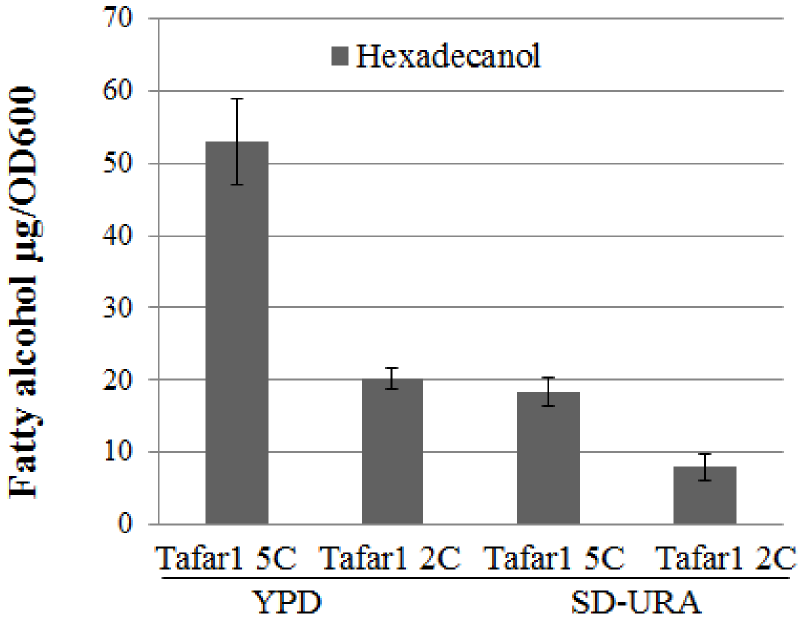

Fig. S2 Fatty alcohol-producing capability of engineered strains on enriched medium (YPD)

Fatty alcohol-producing rate was detected after 24h culturing. Results are the mean of duplicate experiments and error bars indicate standard deviations.

Tafar 5C: *Tafar1*-5copy-Δ*dga1 fao1* strain; Tafar 2C: *Tafar1*-2copy-Δ*dga1 fao1* strain

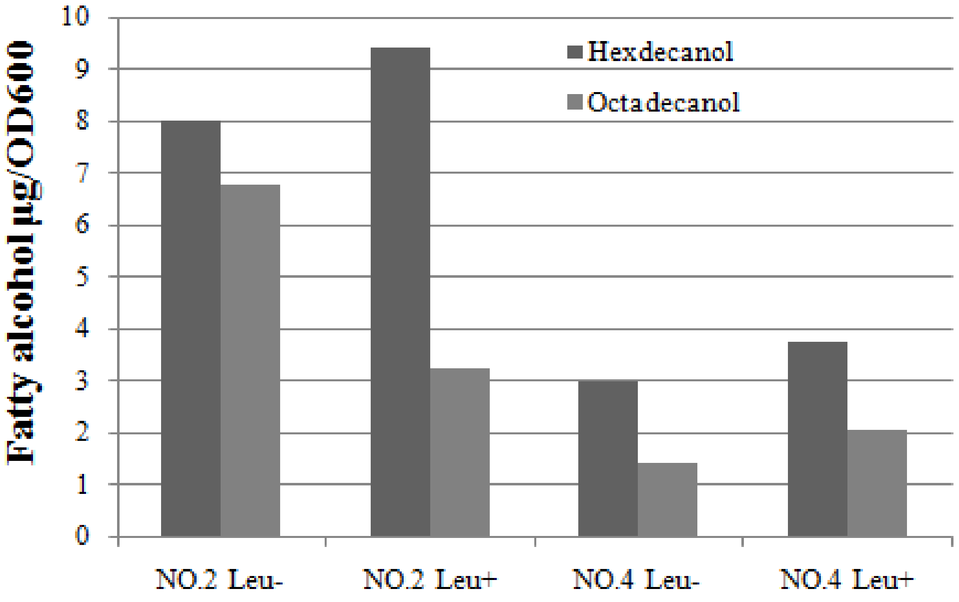

Fig. S3 Limited positive effect of leucine supplement on fatty alcohol production by *Tafar1*-2copy-Δ*fao1* strain

Two *Tafar1*-2copy-Δ*fao1* strains were identified after two-round screening and purification of transformants. Preculture of these strains was used for inoculation into SD-URA (with or without 1.6 g/L leucine supplement, 0.05 of initial OD_{600}) and fatty alcohol-producing rate was detected after 24h culturing.

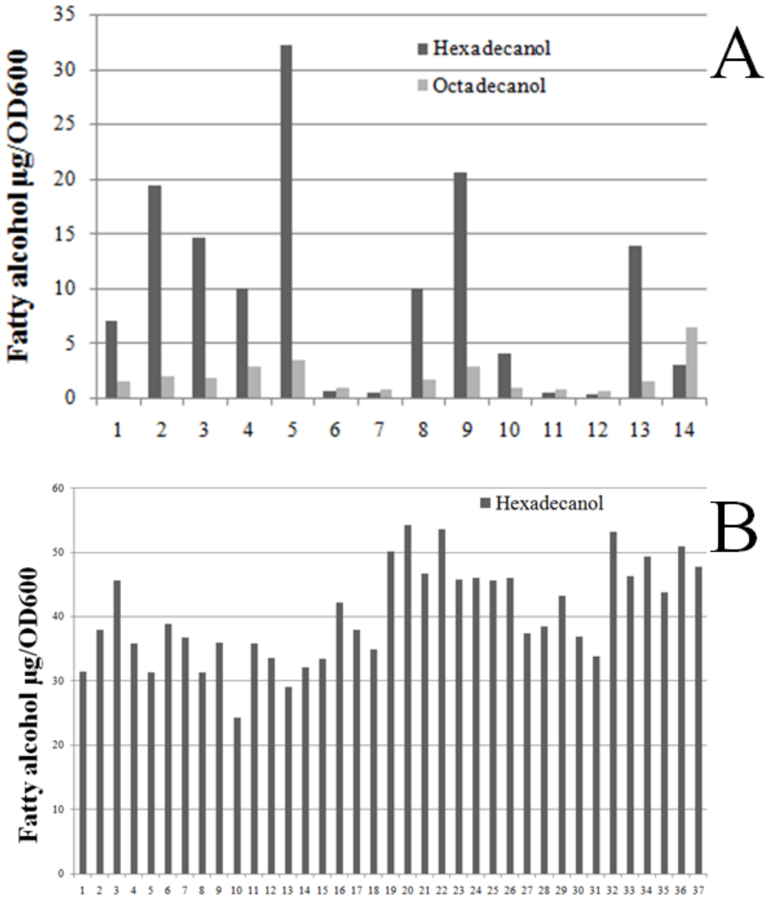

Fig. S4 Two-round screening (A and B) and purification of *Tafar1*-5copy-Δ*dga1 fao1* strains

Transformants of *Tafar1*-2copy-Δ*dga1 fao1* (A) and *Tafar1*-5copy-Δ*dga1 fao1* strains (B, derived from NO. 5 strain of graph A) were used for inoculation into SD-LEU, and fatty alcohol-producing rate was detected after 24h culturing.

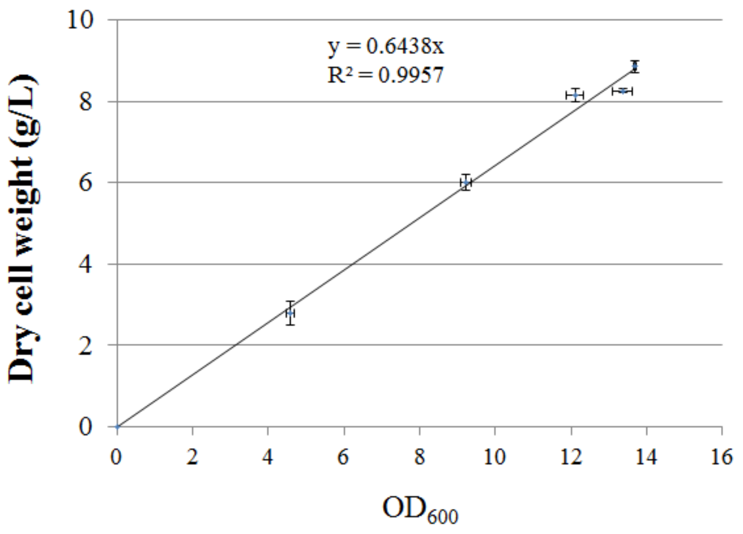

Fig. S5 The correlation between optical density (OD_{600}) and dry cell weight (DCW) of *Y. lipolytica* cells

Tafar1-5copy-Δ*dga1 fao1* strain was subcultured to YPD medium (initial OD 0.05). DCW and OD_{600} were detected over culture time (day 1 to day 5). Results are the mean of duplicate experiments and error bars indicate standard deviations.
