# Supplementary material for: Exploring fatty alcohol-producing capability of Yarrowia lipolytica
Source: Biotechnol Biofuels. 2016 May 20;9:107. doi: 10.1186/s13068-016-0512-3 (PMC4875687; doi:10.1186/s13068-016-0512-3)
Supplement: Supplementary file 2 — 10.1186/s13068-016-0512-3 Demonstration of growth retardation from fatty alcohol/aldehyde accumulation in 24 h. Table S2. Strains used in this study. Table S3. Primers used in this study. [file 13068_2016_512_MOESM2_ESM.docx]

Table S1 Demonstration of growth retardation from fatty alcohol/aldehyde accumulation in 24 h subcultured cells

| Strain | OD_600_ | |  |
| --- | --- | --- | --- |
| Po4f uracil+ leucine+ JN34 Epi | | 5.16±0.11 | |
| *fao1* uracil+ leucine+ JN34 Epi | | 4.82±0.10 | |
| *fao1* uracil+ leucine+ Tafar1 Epi | | 3.40±0.21 | |
| *dga1 fao1* uracil+ leucine+ Tafar1 Epi | | 2.57±0.34 | |
| *dga1 fao1* uracil+ Tafar1-2 leucine+ JN34 Epi | | 1.54±0.20 | |
| *dga1 fao1* uracil+ Tafar1-2 leucine+ Tafar1-3 | | 1.29±0.03 | |

Table S2 Strains used in this study.

| Strains | Genotype | Comments |
| --- | --- | --- |
| Po1f | MatA, leucine-, uracil-, xpr2-322, axp1-2 |  |
| Po4f | MatA, leucine-, uracil-, xpr2-322, axp1-2, *ku70* |  |
| Po4f uracil- leucine+ JN34 Epi | MatA, leucine+, uracil-, xpr2-322, axp1-2, *ku70*, P_TEF_*-*Txpr2 | Fatty acyl-CoA reductase screening |
| Po4f uracil- leucine+ Mafar Epi | MatA, leucine+, uracil-, xpr2-322, axp1-2, *ku70*, P_TEFin_-*Mafar*-Txpr2 |  |
| Po4f uracil- leucine+ Jojobafar1 Epi | MatA, leucine+, uracil-, xpr2-322, axp1-2, *ku70*, P_TEFin_-*Jojobafar1*-Txpr2 |  |
| Po4f uracil- leucine+ Atfar1 Epi | MatA, leucine+, uracil-, xpr2-322, axp1-2, *ku70*, P_TEF_*-Atfar1-*Txpr2 |  |
| Po4f uracil- leucine+ Atfar6 Epi | MatA, leucine+, uracil-, xpr2-322, axp1-2, *ku70*, P_TEF_-*Atfar6*-Txpr2 |  |
| Po4f uracil- leucine+ Mfar1 Epi | MatA, leucine+, uracil-, xpr2-322, axp1-2, *ku70*, P_TEFin_-*Mfar1*-Txpr2 |  |
| Po4f uracil- leucine+ Tafar1 Epi | MatA, leucine+, uracil-, xpr2-322, axp1-2, *ku70*, P_TEFin_-*Tafar1*-Txpr2 |  |
|  |  |  |
| H222-S4 Tafar1 Epi | *MATA ura3-302::SUC2,* P_TEF_-*Tafar1-*Txpr2 uracil+ | Effect assessment of degradation pathway |
| H222ΔP Tafar1 Epi | *MATA ura3-302::SUC2, pox1*, *pox2, pox3, pox4, pox5, pox6,* P_TEF_*-Tafar1*-Txpr2 uracil+ |  |
| H222ΔPΔF Tafar1 Epi | *MATA ura3-302::SUC2, pox1*, *pox2, pox3, pox4, pox5, pox6, fao1,* P_TEF_*-Tafar1*-Txpr2 uracil+ |  |
| H222ΔPΔA Tafar1 Epi | *MATA ura3-302::SUC2, pox1*, *pox2, pox3, pox4, pox5, pox6,fadh, adh1, adh2, adh3, adh4, adh5, adh6, adh7,* P_TEF_*-Tafar1*-Txpr2 uracil+ |  |
| H222ΔPΔAΔF Tafar1 Epi | *MATA ura3-302::SUC2, pox1*, *pox2, pox3, pox4, pox5, pox6,fadh, adh1, adh2, adh3, adh4, adh5, adh6, adh7, fao1,* P_TEF_*-Tafar1*-Txpr2 uracil+ |  |
| Po4f uracil+ leucine+ Tafar1 Epi | MatA, leucine+, uracil+, xpr2-322, axp1-2, *ku70*, P_TEF_-*Tafar1*-Txpr2, *ura3* |  |
| *fao1* uracil+ | MatA, leucine-, uracil+, xpr2-322, axp1-2, *ku70*, *fao1* |  |
| *fao1* uracil+ leucine+ JN34 Epi | MatA, leucine+, uracil+, xpr2-322, axp1-2, *ku70*, *fao1*, P_TEF_*-*Txpr2 |  |
| *fao1* uracil+ leucine+ Tafar1 Epi | MatA, leucine+, uracil+, xpr2-322, axp1-2, *ku70*, *fao1*, P_TEF_-*Tafar1*-Txpr2 |  |
|  |  |  |
| Po4f uracil+ leucine+ Tafar1 Epi | MatA, leucine+, uracil+, xpr2-322, axp1-2, *ku70*, P_TEF_-*Tafar1*-Txpr2, *ura3* | Fatty acyl-CoA supply |
| *pxa2* uracil+ leucine+ Tafar1 Epi | MatA, leucine+, uracil+, xpr2-322, axp1-2, ku70, *pxa2*, P_TEF_-*Tafar1*-Txpr2 |  |
| *pex10* uracil+ leucine+ Tafar1 Epi | MatA, leucine+, uracil+, xpr2-322, axp1-2, *ku70*, *pex10*, P_TEF_-*Tafar1*-Txpr2 |  |
| *dga1* uracil+ leucine+ Tafar1 Epi | MatA, leucine+, uracil+, xpr2-322, axp1-2, *ku70*, *dga1*, P_TEF_-*Tafar1*-Txpr2 |  |
| *dga1 dga2 lro1* leucine+ uracil+ Tafar1 Epi | MatA, leucine+, uracil+, xpr2-322, axp1-2, *ku70*, *dga1, dga2, lro1,* P_TEF_-*Tafar1*-Txpr2 |  |
| *dga1 dga2 lro1 are1* leucine+ uracil+ Tafar1 Epi | MatA, leucine+, uracil+, xpr2-322, axp1-2, *ku70*, *dga1, dga2, lro1, are1,* P_TEF_-*Tafar1*-Txpr2 |  |
| *fao1* uracil+ Tafar1-2 leucine- | MatA, leucine+, uracil+, xpr2-322, axp1-2, *ku70*, *fao1*, P_TEF_-*Tafar1* 2copy-Txpr2 |  |
| *fao1* uracil+ Tafar1-2 leucine+ JN34 Epi | MatA, leucine+, uracil+, xpr2-322, axp1-2, *ku70*, *fao1*, P_TEF_-*Tafar1* 2copy-Txpr2, P_TEF_-Txpr2 |  |
| *fao1* uracil+ Tafar1-2 leucine+ ACC Epi | MatA, leucine+, uracil+, xpr2-322, axp1-2, *ku70*, *fao1*, P_TEF_-*Tafar1* 2copy-Txpr2, P_TEFin_-*Ylacc1*-Tlip1 |  |
| *fao1* uracil+ Tafar1-2 leucine+ ACL Epi | MatA, leucine+, uracil+, xpr2-322, axp1-2, *ku70*, *fao1*, P_TEF_-*Tafar1* 2copy-Txpr2, P_GPM_*-Ylacl1*-Toct1, P_FBA_-*Ylacl2*-Tlip1 |  |
| *fao1* uracil+ Tafar1-2 leucine+ ACS Epi | MatA, leucine+, uracil+, xpr2-322, axp1-2, *ku70*, *fao1*, P_TEF_-*Tafar1* 2copy-Txpr2, P_GPM_-*Scacs1*-Toct1 |  |
| *fao1* uracil+ Tafar1-2 leucine+ FAA Epi | MatA, leucine+, uracil+, xpr2-322, axp1-2, *ku70*, *fao1*, P_TEF_-*Tafar1* 2copy-Txpr2, P_FBA_-*Ylfaa1*-Tlip1 |  |
|  |  |  |
| *fao1* uracil+ leucine+ Tafar1 Epi | MatA, leucine+, uracil+, xpr2-322, axp1-2, *ku70*, *fao1*, P_TEF_-*Tafar1*-Txpr2 | Effect of *Tafar1* expression level |
| *fao1* uracil+ leucine+ Tafar1-2 Epi | MatA, leucine+, uracil+, xpr2-322, axp1-2, *ku70*, *fao1*, P_TEF_-*Tafar1*-*Tafar1* 2copy-Txpr2 |  |
| *fao1* uracil+ leucine+ Tafar1-3 Epi | MatA, leucine+, uracil+, xpr2-322, axp1-2, *ku70*, *fao1*, P_TEF_-*Tafar1*-*Tafar1* 3copy-Txpr2 |  |
| *fao1* uracil+ Tafar1-2 leucine+ JN34 Epi | MatA, leucine+, uracil+, xpr2-322, axp1-2, *ku70*, *fao1*, P_TEF_-*Tafar1* 2copy-Txpr2, P_TEF_-Txpr2 |  |
| *fao1* uracil+ Tafar1-2 leucine+ Tafar1 Epi | MatA, leucine+, uracil+, xpr2-322, axp1-2, *ku70*, *fao1*, P_TEF_-*Tafar1* 2copy-Txpr2, P_TEF_-*Tafar1*-Txpr2 |  |
| *fao1* uracil+ Tafar1-2 leucine+ Tafar1-2 Epi | MatA, leucine+, uracil+, xpr2-322, axp1-2, *ku70*, *fao1*, P_TEF_-*Tafar1* 2copy-Txpr2, P_TEF_-*Tafar1* 2copy-Txpr2 |  |
| *fao1* uracil+ Tafar1-2 leucine+ Tafar1-3 Epi | MatA, leucine+, uracil+, xpr2-322, axp1-2, *ku70*, *fao1*, P_TEF_-*Tafar1* 2copy-Txpr2, P_TEF_-*Tafar1* 3copy-Txpr2 |  |
|  |  |  |
| *dga1 fao1* uracil+ leucine+ Tafar1 Epi | MatA, leucine+, uracil+, xpr2-322, axp1-2, *ku70*, *dga1, fao1,* P_TEF_-*Tafar1*-Txpr2 | Combinatorial improvement |
| *dga1 dga2 lro1* *fao1* uracil+ leucine+ Tafar1 Epi | MatA, leucine+, uracil+, xpr2-322, axp1-2, *ku70*, *dga1, dga2, lro1, fao1,* P_TEF_-*Tafar1*-Txpr2 |  |
| *dga1 fao1* uracil+ Tafar1-2 leucine- | MatA, leucine-, uracil+, xpr2-322, axp1-2, *ku70*, *dga1, fao1,* P_TEF_-*Tafar1* 2copy-Txpr2 |  |
| *dga1 fao1* uracil+ Tafar1-2 leucine+ Tafar1-3 | MatA, leucine-, uracil+, xpr2-322, axp1-2, *ku70*, *dga1, fao1,* P_TEF_-*Tafar1* 2copy-Txpr2, PTEF-*Tafar1* 3copy-Txpr2 |  |

Table S3 Primers used in this study.

| \| Primer \| Sequence \| Comment \| \| --- \| --- \| --- \| \| Mafar-F \| TTAAACATATGATGAATTATTTCCTGACAGGCGG \| Cloning of genes coding fatty acyl-CoA reductases \| \| Mafar-R \| AATTTGTCGACTTACCAGTATATCCCCCGCATAA \| \| Scfar-F \| CGCTTCCCGGGATGGAAGAAATGGGATCAAT \| \| Scfar-R \| CGCTTCCCGGGTCAATTAAGAACATGCTCAA \| \| Atfar1-F \| GGCCCCTGCAGATGGAATCCAATTGTGTTCA \| \| Atfar1-R \| CGCTTCCCGGGTTATTGTTTAAGCACATGGG \| \| Atfar6-F \| GGCCCCTGCAGATGTGTTTTTATGGTGAGAC \| \| Atfar6-R \| CGCTTCCCGGGTTACTCAGTCTTCTTCTTAG \| \| mfar1-F \| GGCCCAAGCTTATGGTGAGCATCCCAGAGTA \| \| mfar1-R \| CGCTTCCCGGGTTAGTAGCGCATGGTGGAGG \| \|  \|  \|  \| \| FAO1-5F \| ATTAAGCGGCCGCGTGCTCTAGTCTCGGTGGCG \| Flanking region obtainment for gene deletion \| \| FAO1-5R \| CCTAGTCTAGAGCGAAGCGACGTGTGGTGAG \| \| FAO1-3F \| ATTGGACTAGTGCTGAGCACGCGAGTACACC \| \| FAO1-3R \| AAGTTCATATGGTGAGTCTGGCATCCATTAA \| \|  \|  \| \| PXA2-5F \| GTATTGGGCCCATATCGGTTCCCTTTCA \| \| PXA2-5R \| GCGCTTCTAGAGACGTTTGCCATGATG \| \| PXA2-3F \| CGATTGGATCCAAGAAGCGAAAGACCGA \| \| PXA2-3R \| CGCATCTGCAGACACTCTTCATGTATCC \| \|  \|  \| \| PEX10-5F \| CGATTGGGCCCATAACCTCCATGTACGAT \| \| PEX10-5R \| GTTTTTCTAGAGCCGAGGCAGATTTGGG \| \| PEX10-3F \| TGACGAGGTCTGGATGGAAGGACTA \| \| PEX10-3R \| GCCTACATATGACCAGATCAGACGCCCAA \| \|  \|  \| \| DGA1-5F \| CGAACGGGCCCTGGTGCATTTTTGCTTGCGAT \| \| DGA1-5R \| CGAACTCTAGATGGGAGCTTATCAGTCACGG \| \| DGA1-3F \| CTATTACTAGTGGAAAACTGCCTGGGTTAGG \| \| DGA1-3R \| CAACTAGTAGATGACCCTGACGCAGATG \| \|  \|  \| \| DGA2-5F \| ATGCAGCATGCTATCTTCCCACGTTTGTATA \| \| DGA2-5R \| GCTATTCTAGAACGACTATGAGCAAGCCTGA \| \| DGA2-3F \| CGCTTACTAGTAAGCACGTGATCCGAAACCT \| \| DGA2-3R \| CCTGTCATATGTCGCCACGATCTGTACTCCT \| \|  \|  \| \| LRO1-5F \| CGATCGGGCCCGGAACCAGACTTGCTCCACA \| \| LRO1-5R \| CGATATCTAGAACCGGGGTAGCTGAGACAT \| \| LRO1-3F \| GCATAACTAGTCAATCCCGAGCAGACCAACT \| \| LRO1-3R \| ACGTAACTAGTACCCTGGAATGTGAAGCGAG \| \|  \|  \| \| ARE1-5F \| TTACGGCATGCAGCAATACGGTTCCTGGACG \| \| ARE1-5R \| GTACGTCTAGACTCCCGCACTATCGAGTGTC \| \| ARE1-3F \| GCCATACTAGTTCTGGGACGCCATTCTCAAC \| \| ARE1-3R \| GGGATCATATGGACCGCCGTGCTAAAAAGAC \| \|  \|  \|  \| \| ACC1-F \| GCCATCCCGGGCGACTGCAATTGAGGACACT \| Gene cloning for driving metabolic flux to fatty acyl-CoA \| \| ACC1-R \| CCCTTCCCGGGTCACAACCCCTTGAGCAGCT \| \| ACL1-F \| GGCCCAAGCTTATGTCTGCCAACGAGAACAT \| \| ACL1-R \| CGCTTCCCGGGCTATGATCGAGTCTTGGCCT \| \| ACL2-F \| GGCCCAAGCTTATGTCAGCGAAATCCATTCA \| \| ACL2-R \| GGCCCAAGCTTTTAAACTCCGAGAGGAGTGG \| \| SCACS1-F \| GGCCCAAGCTTATGTCGCCCTCTGCCGTACA \| \| SCACS1-R \| CGCTTCCCGGGTTACAACTTGACCGAATCAA \| \| FAA1-F \| CGCTTCCCGGGATGGTCGGATACACAATTTC \| \| FAA1-R \| CGCTTCCCGGGCTAAGACTGCTCGTAGCACT \| \|  \|  \|  \| \| ACTIN-F \| TCCAGGCCGTCCTCTCCC \| Real time PCR \| \| ACTIN-R \| GGCCAGCCATATCGAGTCGCA \| \| Tafar1-RT-F \| CCGACCCAACACCTACAC \| \| Tafar1-RT-R \| GGTCCGTTAAAGTTGTCAATC \| |  |  |
| --- | --- | --- | --- | --- | --- | --- | --- | --- | --- | --- | --- | --- | --- | --- | --- | --- | --- | --- | --- | --- | --- | --- | --- | --- | --- | --- | --- | --- | --- | --- | --- | --- | --- | --- | --- | --- | --- | --- | --- | --- | --- | --- | --- | --- | --- | --- | --- | --- | --- | --- | --- | --- | --- | --- | --- | --- | --- | --- | --- | --- | --- | --- | --- | --- | --- | --- | --- | --- | --- | --- | --- | --- | --- | --- | --- | --- | --- | --- | --- | --- | --- | --- | --- | --- | --- | --- | --- | --- | --- | --- | --- | --- | --- | --- | --- | --- | --- | --- | --- | --- | --- | --- | --- | --- | --- | --- | --- | --- | --- | --- | --- | --- | --- | --- | --- | --- | --- | --- | --- | --- | --- | --- | --- | --- | --- | --- | --- | --- | --- | --- | --- | --- | --- | --- |
|  |  |  |
|  |  |  |
